# Impact of an exergame intervention on cognitive-motor functions and training experience in young team sports athletes: a non-randomized controlled trial

**DOI:** 10.3389/fspor.2023.1170783

**Published:** 2023-11-24

**Authors:** Anna Lisa Martin-Niedecken, Valentin Bucher, Manuela Adcock, Eling D. de Bruin, Alexandra Schättin

**Affiliations:** ^1^ Department of Design, Institute for Design Research, Zurich University of the Arts, Zurich, Switzerland; ^2^ Exergame Research Hub, Sphery Ltd., Zurich, Switzerland; ^3^ Department of Health Sciences and Technology, Institute of Human Movement Sciences and Sport, ETH Zurich, Zurich, Switzerland; ^4^ Department of Health, OST – Eastern Swiss University of Applied Sciences, St. Gallen, Switzerland; ^5^ Division of Physiotherapy, Department of Neurobiology, Care Sciences and Society, Karolinska Institute, Stockholm, Sweden

**Keywords:** exergame, training intervention, cognitive-motor functions, executive functions, team sports athletes, performance

## Abstract

**Introduction:**

Team sports athletes need excellent perceptual-cognitive skills, particularly executive functions (EF) to strategically perform on the field. The transfer effect of cognitive training might be accomplished by the inclusion of cognitive stimuli into a physically active environment as these couplings are required in real game situations. A training approach that combines both components is exergaming. The primary objective of this study was to gain preliminary insights into the effects of exergaming on cognitive-motor functions in young team sports athletes. The secondary objective was to investigate participants' training experience and well-being over time.

**Methods:**

Participants were assigned to the intervention or control group. In the intervention group, participants trained with the ExerCube—a mixed reality exergame. The training was planned for 10 weeks (two sessions per week à 25 min) but had to be shortened by 2 weeks due to COVID-19 restrictions. The control group had no additional training. Outcomes included a computer-based alertness test and a cognitive-motor test battery to assess different EF (flexibility, divided attention, and inhibition) via a FitLight Trainer setup.

**Results:**

Twenty-four athletes [mean age (±SD) 15.0 ± 0.7 years], evenly split into the intervention group (*N* = 12; male *N* = 6; female *N* = 6; 14.7 ± 0.5 years) and the control group (*N* = 12; male *N* = 7; female *N* = 5; 15.3 ± 0.8 years), participated in the study. Participants in the intervention group performed on average 9.4 ± 3.3 training sessions over 8 weeks. Significant time x group interaction effects were evident for the cognitive-motor sub-tests flexibility (*F* = 12.176, *p* < 0.001, *d* = 1.488) and divided attention for auditive stimuli (*F* = 9.776, *p* = 0.002, *d* = 1.404) in favour of the intervention group. For the alertness test, a medium effect size (time x group interaction) was seen for the variability of the reaction time (*F* = 2.196, *p* = 0.138, *d* = 0.632) in favour of the intervention group. Training experience and well-being were consistently at medium to high levels.

**Conclusion:**

The ExerCube training yielded positive effects on concentration, flexibility, and divided attention indicating that exergaming can be an innovative training approach for team sports athletes.

## 1. Introduction

In competitive team sports, athletes require (extraordinary) perceptual-cognitive skills (1). Particularly, athletes performing in dynamic team sports must have great cognitive abilities to process much information in a short time leading to a situation appropriate action response in a rapidly and constantly changing environment (2). Proper executive functions (EF) allow flexible action planning and behaviour adaption to constantly changing environments (3, 4) such as strategic explorative behaviour in team sports (5). Fast reaction, inhibitory skills, flexibility, and divided attention are important for those athletes to succeed on the field (6–9). Furthermore, so-called general EF, including on-line multi-processing such as creativity, response inhibition, and cognitive flexibility, are deciding team sport success in performance (10, 11). Thus, well-developed cognitive functions, and especially EF, are important to strategically perform on the field in challenging team sports (10, 12).

Consequently, increased interest exists in successful forms of EF training that lead to improved athletic performance on the field, and there is a need for more research on this topic (13). Studies demonstrated that physical exercising, especially aerobic exercise, has positive effects on EF (14–17). Next to physical exercising also (perceptual-)cognitive training approaches using computerized methods, e.g., video gaming, seem to beneficially affect EF (18–20). Nevertheless, researchers promote the simultaneous combination of physical and cognitive exercise as the positive effects seem to be additive in terms of cognitive functioning (21, 22).

In terms of transfer effects of EF, (perceptual-)cognitive training approaches revealed contradictory findings. Certain results indicate some improvements in skills of soccer players (23) whereas others report lack of broad transfer effects (24, 25). Furthermore, the transfer effects of cognitive training in team sports athletes might be increased by the inclusion of cognitive stimuli into a physically active environment as these couplings are required and highly challenged in real game situations (26, 27). A recent study observed that changes in brain activation and functional connection were more rapidly induced by physical and cognitive fatigue compared to mental fatigue (28).

A promising training approach that concurrently combines cognitive and physical training is video game-based physical exercise, so-called exergaming. Exergames (29) are “technology-driven physical activities, such as video game play, that require participants to be physically active or exercise in order to play the game” (30).

Studies using exergames showed enhanced cognitive functioning, especially executive control skills, in younger populations (31–34). Benzing et al. indicated that acute exergame-based physical activity with high cognitive load seems to be more effective than just physical activity of the same intensity in improving cognitive flexibility (31). Anzeneder et al. showed that an exergame-based acute 15 minutes (min) cognitively high-challenging bout of physical exercise enhances allocable resources in children, which in turn facilitate information processing, and executive processes (35). However, the current evidence is based on acute effects, and further studies are needed to investigate the long-term effects of exergaming in adolescents.

Regarding potential sports specific transfer effects, integrating exergames into a regular tennis training program revealed beneficial effects on cognitive-motor tennis skills by adding dynamics to the athletes training regime (36). Thus, exergaming may provide a transfer of athletic skills to sports activities (34). Moreover, playing exergames can have a positive effect on psychosocial outcomes as social interaction, self-esteem, mood, and motivation (34). However, evidence is lacking how an additional holistic exergame training may influence cognitive-motor functions (e.g., EF) in young team sports athletes (2). To increase possible transfer effects, an exergame involving cognitive stimuli in a functional whole-body training environment seems most promising (37).

The ExerCube, designed by sports scientists and game designers, offers a whole-body functional training carried out in a cognitively engaging mixed reality game setting (38). Previous studies showed that the ExerCube provides a form of vigorous physical exercise in a joyful, immersive, and motivational gaming environment that can be adapted to individual needs and requirements (38–40). The primary objective of this study was to get preliminary insights into the effects of an ExerCube training intervention on cognitive-motor functions in young team sports athletes. The secondary objective was to investigate participants' training experience (enjoyment, motivation, and flow) as well as mental well-being.

## 2. Method

### 2.1. Study design and procedure

This study was a non-randomized controlled trial examining the training effects of an ExerCube training intervention on cognitive-motor functions, training experience, and mental wellbeing in young team sports athletes. The study ran from January to March 2020. Training intervention and measurements were performed at the Win4 Campus (Winterthur, Switzerland, https://win-4.ch/).

Participants were either allocated to the intervention group or the control group. Participants in the intervention group performed an ExerCube training twice per week for 25 min. The training intervention was planned to last 10 weeks, as similar studies have found training effects in 5 (41) to 10 (42) weeks using traditional cognitive-motor training approaches. Due to unexpected COVID-19 restrictions, the study intervention period had to be terminated prematurely by 2 weeks, and the post-intervention measurements were brought forward. The first two ExerCube training sessions were supervised by a study investigator educated in the ExerCube application, thus, allowing familiarisation of the participants with the exergame system and detailed instruction of use. For the following training sessions, participants performed the ExerCube training independently. If participants had any issues with the system, they had the possibility to contact a responsible and ExerCube-educated person on site. Participants had to attend at least 70% of the training sessions to be included in the analysis, as this participation rate was found to be appropriate in comparable studies on cognitive-motor effects of exergame training (43). Furthermore, it was foreseeable that it would not always be possible for the athletes to participate in all the planned training sessions or for an even longer total duration of the intervention due to other training commitments and pre-competition and post-competition preparation. Participants in the control group had no additional (exergame) training sessions and were instructed to continue their daily activities.

Measurements of cognitive-motor functions and mental wellbeing were performed at pre- and post-intervention measurements in both groups. Measurements about training experience were performed after the first training session and at the post-intervention measurement, and were only assessed in the intervention group. Baseline data were assessed at pre-intervention measurement via a questionnaire. Measurements were executed and supervised by an educated study investigator.

The study was registered at ClinicTrials.gov (NCT04296708). TREND checklist was used for the reporting of this trial (44).

### 2.2. Participants

For this study, young team sports athletes from top-level sports promotion, playing ice hockey, floorball, soccer, or handball, were included. They had to be between 14 and 20 years old, healthy (self-reported via baseline questionnaire), able to provide written informed consent, and understand the study instructions in German. Exclusion criteria were: (1) cardiovascular issues and musculoskeletal injuries which would prevent training participation, (2) pain which would be reinforced by sportive activities, (3) uncontrollable asthma, (4) epilepsy, and (5) pregnancy. The intended sample size was set at 30 participants and based on an estimation of availability for convenience sampling as this is an explorative study that provides initial insights into the impact of exergaming in highly skilled young team sports athletes.

Team sports athletes (Win4 Campus Winterthur, Zurich, Switzerland) were informed by word of mouth by their coaches. Coaches were informed by the study investigators about the study aim, procedures, risks, benefits, and in- and exclusion criteria. Coaches also had the opportunity to test the exergame setup. All coaches were members of local sports clubs in the city of Winterthur (Canton Zurich, Switzerland). All interested and eligible participants and their legal guardians were fully informed about the study prior to the pre-intervention measurement. The allocation of the participants to the study groups was non-randomized as the decision was done by the coaches who were advised to equally allocate their athletes to the two groups regarding their physical and cognitive team sports skills, or by the study investigators using the skill background information from the coaches. The goal was to have primarily a balanced skill distribution and secondary a balance for age and gender. Random allocation was believed to possibly reduce the effectiveness of the intervention.

### 2.3. Training intervention

**Figure 1:**
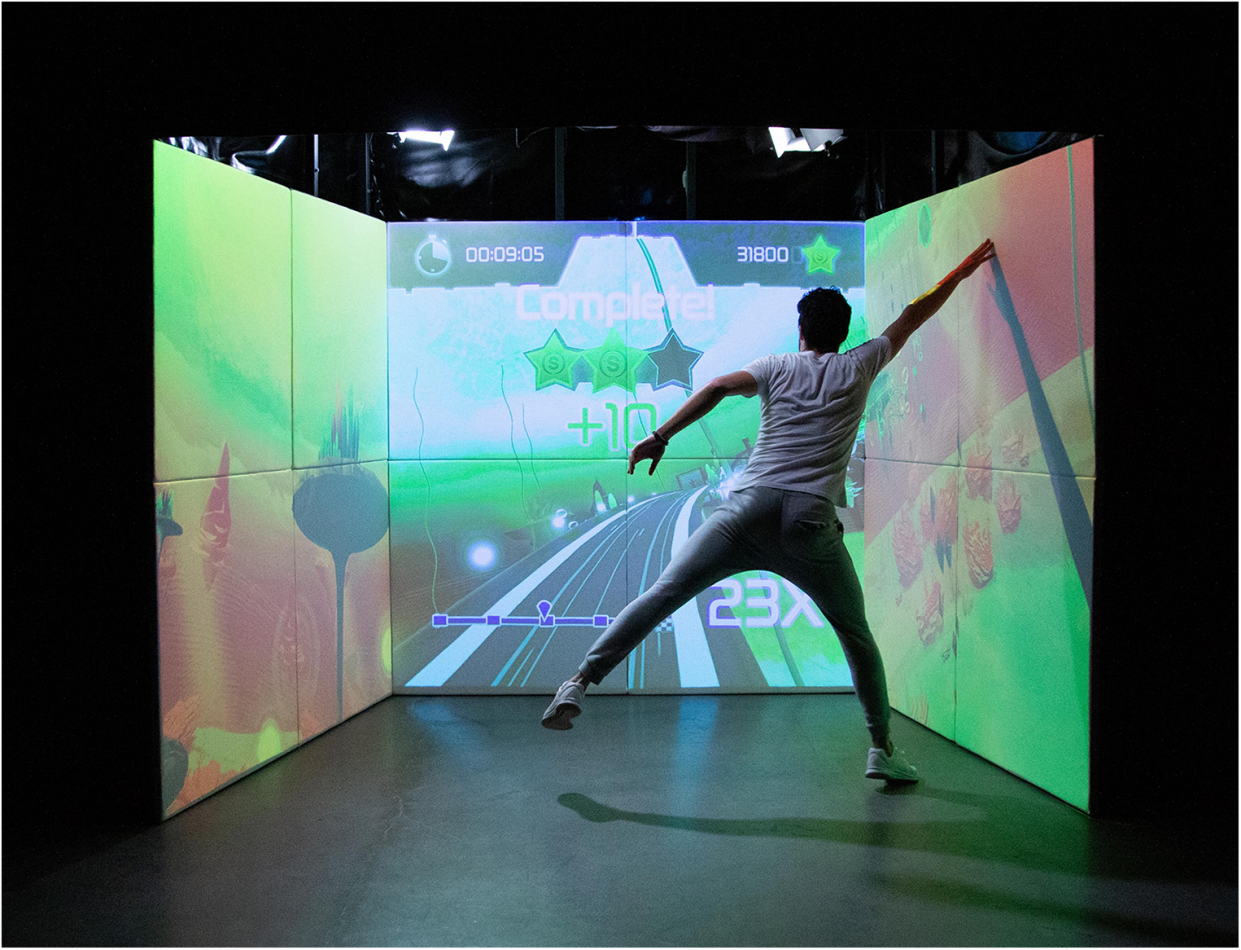
ExerCube training setup © Sphery.

For the purpose of this study, the ExerCube (Sphery AG, Zurich, Switzerland, paris version, v.0.8.0b208/233), an immersive mixed reality fitness game, was used as training stimuli. Three walls (two side and one front wall) serve as project screens and as a haptic interface (Figure 1). The control movements of the fitness game were tracked via HTC Vive trackers which were attached to the wrists and ankles of the player. The ExerCube has been proven to combine important aspects of an attractive (e.g., motivating and enjoyable) and effective (e.g., physical and cognitive demanding) exergame-based training (38–40, 45).

Via functional whole-body exercises, participants controlled an avatar on a virtual underwater racing track (“Sphery Racer”). The game started with low-to-moderate intense exercises and increased over time to high-intensity exercises.

• Interval 1 (2 min): Touch middle, Touch low, Touch high (all touches left and right)
• Interval 2 (3 min): +Squat, Jumping, Punch (all exercises left and right)
• Interval 3 (4 min): +Lunge (all exercises left and right)
• Interval 4 (7 min): +Skipping
• Interval 5 (9 min): +Burpee

Each participant was challenged at the individual fitness and performance level over the intervention period ensuring an ideal workout experience and training progress. The physical challenge was set at 80% of their maximal heart rate (HR_max_) using the following formula (46):

(1) $$HR_{\max}=211\text{-}\mathrm{age}\;x\;0.64$$

## 3. Measurements

### 3.1. Primary outcomes

#### 3.1.1. Test for attentional performance

Test for Attentional Performance (TAP) (D-TAP 2.3 VL, PSYTEST, Psychologische Testsysteme, Herzogenrath, Germany) is a valid test to assess cognitive functions (47). The TAP requires only simple motor responses of the index finger that are executed after specified stimuli. On a personal computer, participants performed the *alertness test* (approx. 4.5 min): Participants had to push the reaction button as fast as possible when a cross appeared on the screen. Each test was preceded by a short pre-test to familiarise and to avoid learning effects. For the analysis, the median of the reaction time (ms), providing information about the general speed of processing, as well as the variability of the reaction time (ms) (standard deviation of reaction time), providing information about the stability of the performance level, were assessed.

#### 3.1.2. Cognitive-motor test battery

**Figure 2:**
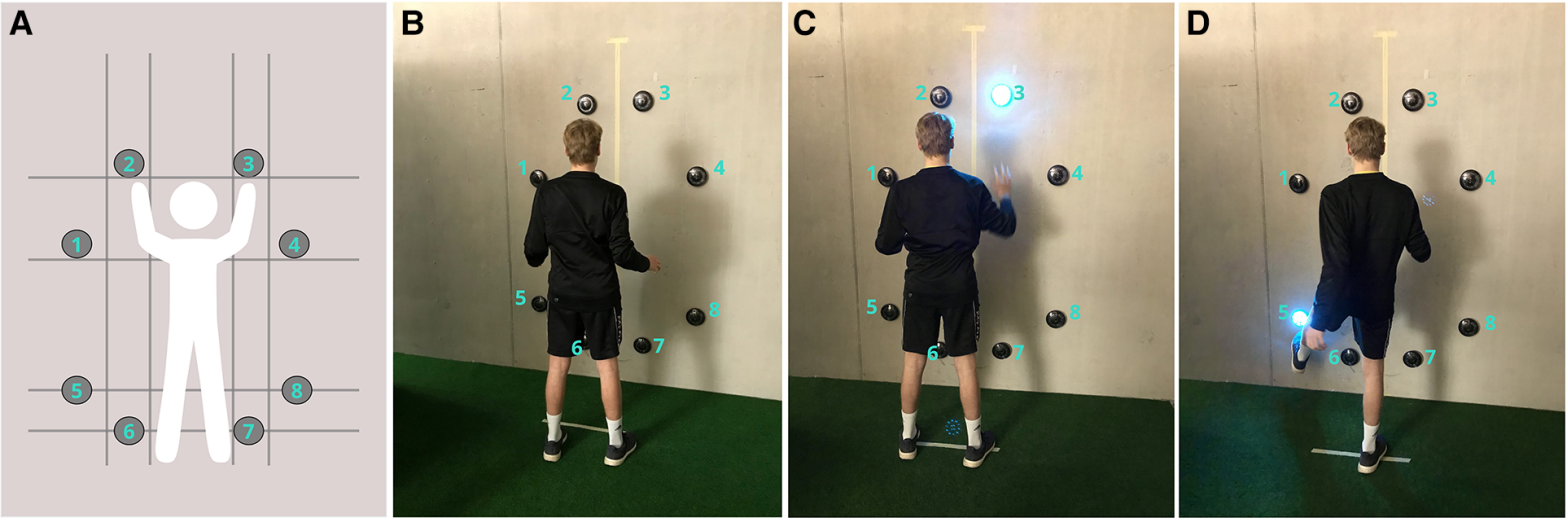
FitLight sensor setup for the cognitive-motor test battery. (**A**) Shows the frontal view of the sensor setup (eight sensors: number 1–8). Position of sensors: 2 and 3 height of the fingertips, 1 and 4 extended line of the upper arm, 5 and 8 height of the patella (middle), 6 and 7 half of the height of the patella. Sensors: 1 and 5, 2 and 6, 3 and 7, and 4 and 8 are on the same vertical line. (**B**) Shows the start and end position after each movement. Sensors 1–4 have to be deactivated with the hands (**C**) and sensors 5–8 have to be deactivated with the feet (**D**). The distance to the wall was defined as the maximal distance that still allows the sensors to be deactivated with the stretched leg. The distance was marked on the floor.

**Figure 3:**
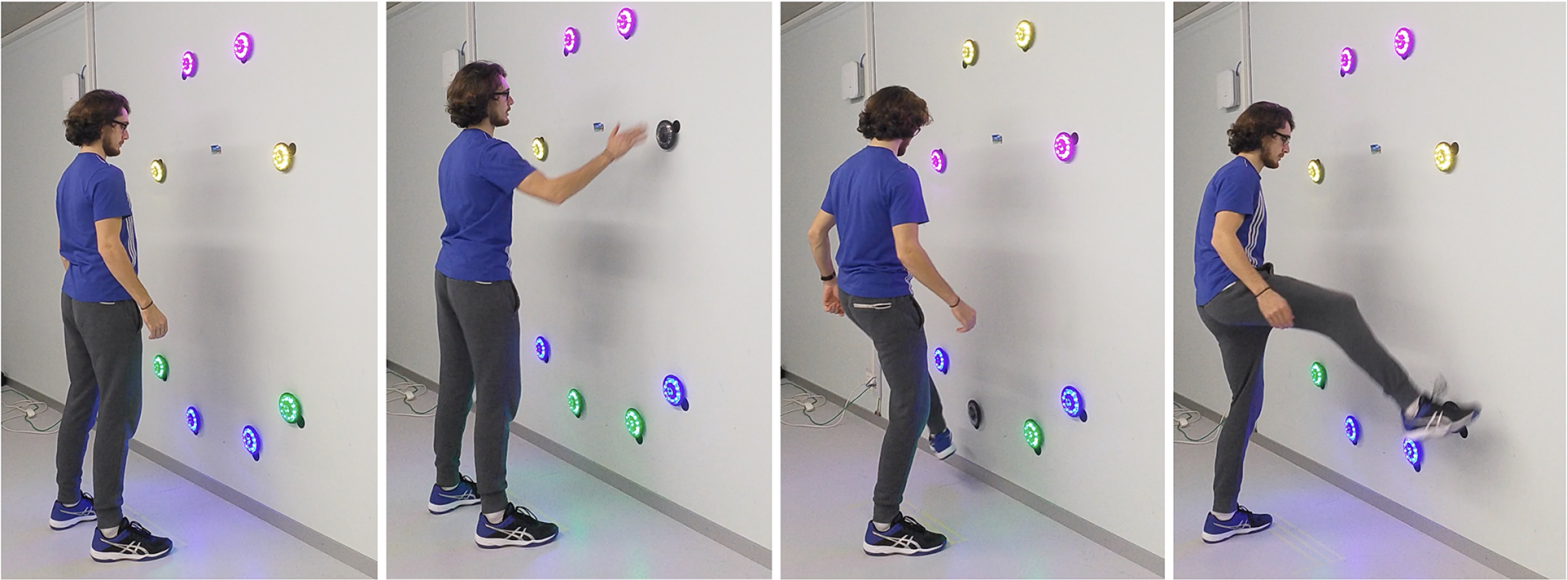
FitLight sensor setup for the flexibility task.

For the cognitive-motor tests, the FitLight Trainer was used (FitLight Sports Corp., Ontario, Canada), a commercially available device that consists of eight wireless LED powered sensors controlled by a tablet (android version 4.2.2) that provides reliable measures of reaction time in healthy adults (48, 49) for cognitive-motor assessment (50). Each sensor can illuminate in different colours, has an inner and an outer LED circle (separate or full illumination), and has the possibility to play a simple audio signal. The lights can be deactivated by touch or proximity. For this project, specific predefined light and audio sequences were used to test different cognitive functions. The four tested cognitive functions are described in the following subchapters. The same stimuli sequences were used for pre- and post-intervention measurements. The sensors were deactivated via hand and foot movements allowing the inclusion of the whole-body movements into the testing scenario. The test setup of the sensors was installed at a wall as illustrated in Figures 2, 3. The setup was adapted for each participant to account for the different body metrics. The sensor positions were noted for each participant at pre-intervention measurement, allowing the same sensor positions for post-intervention measurement. Each test was preceded by a short pre-test sequence allowing familiarisation and minimising learning effects. Furthermore, a specific start sequence (red → yellow → green) was installed. For each test, participants were told to react as fast as possible and to go back to the starting position after each movement. Study investigators intervened if (1) participants crossed the marked line on the floor by more than 10 cm, (2) reached out their hands to the wall, (3) or deactivated a sensor with the wrong extremity. In one of these cases, the stimulus was marked as a failure and was not included in the reaction time analysis. Each measurement session was recorded with two video cameras for analysing purposes.

#### 3.1.3. Cognitive functions

##### 3.1.3.1. Simple reaction time (approx. 1 min 20 s)

The task was to deactivate the illuminated sensor as fast as possible. Only one sensor was activated at a time. The test included 100 stimuli which were randomly distributed over the eight sensors. Presentation time per stimulus was 1.00 s and the interstimulus interval was 0.55 s.

##### 3.1.3.2. Flexibility (approx. 3 min 30 s)

The task was to deactivate the sensors in a specific colour sequence and to alternate between the left and the right side, starting with the right side. The colour sequence was yellow → green → blue. The same colours were presented on the left side (1, 2, 5, and 6) and the right side (3, 4, 7, and 8). The sensors concurrently showed the colour and played a signal tone. It was not allowed to say the colour sequence aloud. If participants made a mistake, they could continue or could restart with yellow on the right side. However, if the colour sequence was wrong, the investigator drew their attention to the correct sequence. The test included 100 stimuli which were randomly distributed over the eight sensors. Presentation time per stimulus was 1.50 s and the interstimulus interval was 0.55 s.

##### 3.1.3.3. Divided attention (approx. 3 min 30 s)

The task was to pay attention to two simultaneous tasks (visual and auditory) and to react to the key stimuli of the two tasks. The sensors on the top (1–4) and bottom (5–8) were alternatively activated, starting with the bottom sensors. When the sensors were activated, the signal tone was concurrently played. The first key stimulus (visual) was if two sensors had the same colour next to each other. The second key stimulus (auditory) was if the signal tone was missing. If an auditory or visual key stimulus was present, participants had to deactivate the green illuminated sensor that acted as a reaction button. If the sensors on the top were activated, the reaction button was active on the bottom and vice versa. Depending on the handedness, sensor 1 or 5 for left-handers or sensor 4 or 8 for right-handers were used as reaction buttons. The test included 100 stimuli of which 17 were auditory key stimuli and 17 were visual key stimuli. Presentation time per stimulus was 1.50 s and the interstimulus interval was 0.55 s.

##### 3.1.3.4. Inhibition (approx. 1 min 45 s)

The task was to inhibit the deactivation of the sensor that was completely (centre and outer ring) dark blue or yellow. If the sensor had a different colour or only the centre ring was illuminated, it had to be deactivated. In this test, only one sensor was illuminated at a time and concurrently the signal tone was played. The test included 60 stimuli (randomly distributed over the eight sensors) of which 24 were key stimuli. Presentation time per stimulus was 1.5 s and the interstimulus interval was 0.55 s.

### 3.2. Secondary outcomes

#### 3.2.1. Questionnaires

The Situational Motivation Scale (SIMS) assessed participants' intrinsic and extrinsic motivation by 16 items (51). The SIMS questionnaire comprises four factors: intrinsic motivation, identified regulation, external regulation, and amotivation. The Flow Short Scale (FSS) was used to evaluate participants' flow experience (52). Ten items measure the flow experience (consisting of the two dimensions “fluency of performance” and “absorption by activity”) whilst three additional items measure “perceived importance”. Further, participants' training enjoyment was assessed via the Physical Activity Enjoyment Scale (PACES), consisting of 18 bipolar statements (53). All three questionnaires were rated on a 7-point (Likert) scale. The Warwick-Edinburgh Mental Well-being Scale (WEMWBS) is a valid and reliable tool for assessing mental well-being by 14 items on a 5-point Likert scale (54, 55). The questionnaire covers the following aspects: positive affect, satisfying interpersonal relationships, and positive functioning. The higher the score is, the higher the level of mental well-being, and a score of 51 can be assumed to be normal (54).

### 3.3. Statistical analysis

For statistical analysis, SPSS 26.0 for Windows (SPSS Inc, Chicago, IL, United States) and RStudio (version 1.2.5042) (56) were used. As the assumptions for parametric statistics were not met, non-parametric tests were used to analyse the data. Baseline data were compared using the Mann-Whitney-*U* test. Pre- and post-intervention measurement data comparison between the groups was analysed using the R package nparLD which was developed for nonparametric analysis of longitudinal data in factorial experiments (57). *Post hoc* analyses were performed using a Wilcoxon signed-rank test for within-group comparisons and a Mann-Whitney-*U* test for between-group comparisons. For all tests, a significance level of *p* ≤ 0.05 was applied. Effect sizes *d* or *r* were calculated with the interpretation to effect sizes d based on benchmarks suggested by Cohen: small (*d* = 0.2), medium (*d* = 0.5), and large (*d* = 0.8) (58). For the effect size *r*, the effect size is low when the value varies around 0.1, medium around 0.3, and large when the values are of 0.5 and above (58).

## 4. Results

**Figure 4:**
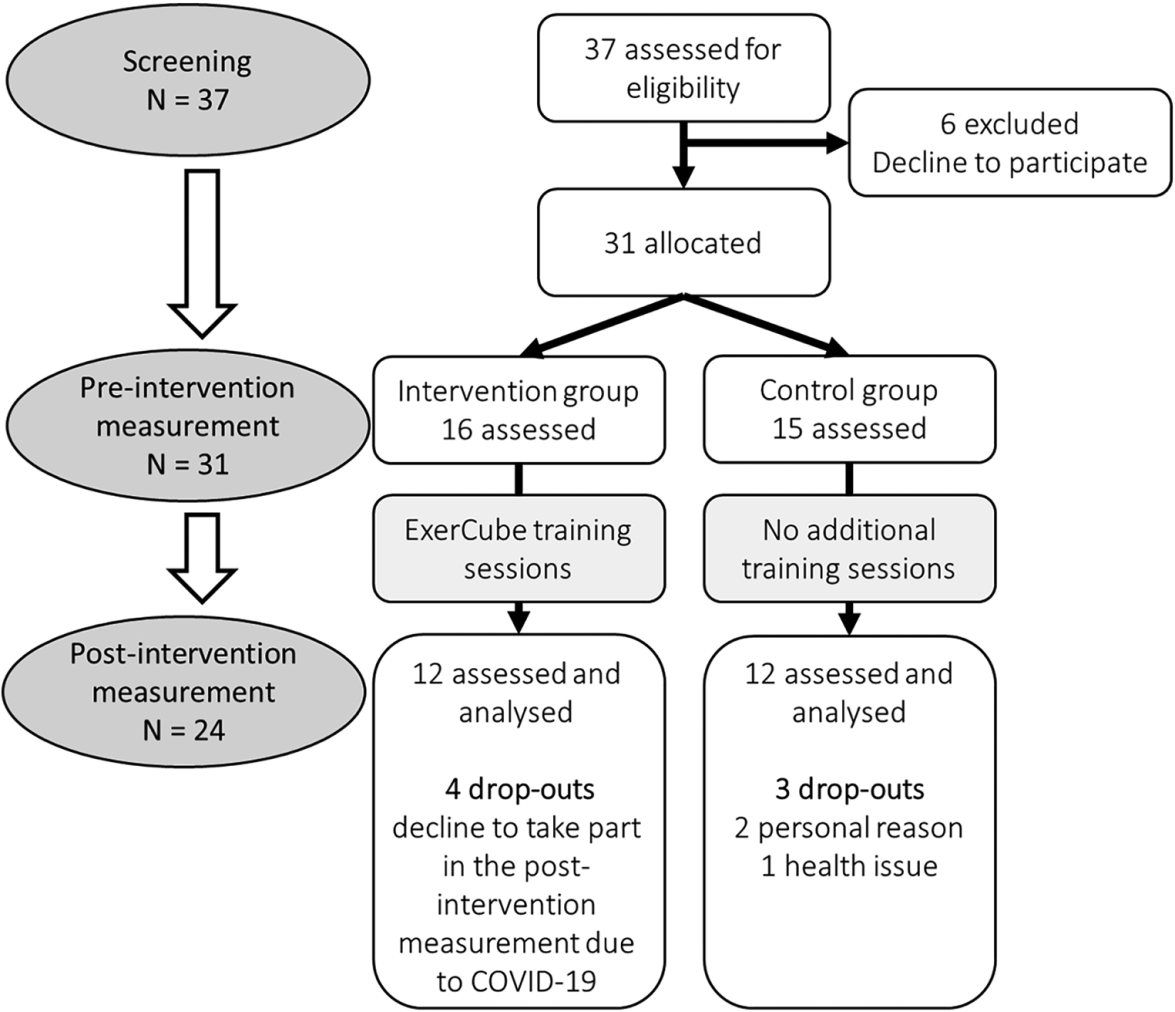
Study flow diagram.

Due to the COVID-19 pandemic, the study had to be terminated before the participants could complete their scheduled training sessions. Only two participants completed 70% of the training sessions (13 training sessions). Therefore, analysis was performed with all participants to still get preliminary insights into the effect of the exergame training on the cognitive-motor functioning in young athletes. Overall, 113 training sessions (median [IQR]: 10.00 [6.75; 11.25]) were performed. Seven participants dropped-out of the study (2 participants due to personal reasons, 1 participant due to health issues, 4 participants could not attend the post-intervention measurements due to COVID-19). The study flow diagram is illustrated in Figure 4. The baseline data of the participants are presented in Table 1. In the intervention group, none of the participants had previous experience with the ExerCube setup. In the control group, one participant had previous experience with exergames and in the intervention group six individuals had previously played an exergame. Regarding general gaming experience, six participants in the control group played video games and four people in the intervention group. No adverse events were recorded for this study.

**Table 1:** Baseline data of the participants.

	Intervention group	Control group	*p*
*N*	12 (6 female)	12 (5 female)	
Team sports [*N*]			
Ice hockey	3	2	
Floorball	5	5	
Football	2	3	
Handball	2	2	
Age [year]	15.0 [14.0; 15.0]	15.0 [15.0; 16.0]	0.068
Education [year]	9.0 [8.3; 9.0]	10.0 [9.0; 10.0]	0.114
Sports activity in team sports [min/per week]	510 [428; 608]	420 [360; 585]	0.233
Fitness evaluation (self-reported)	5.0 [4.3; 5.0]	5.0 [4.3; 5.0]	0.922

*N* = 24. Values are shown as median values [interquartile range]. Group comparisons were analysed using the Mann-Whitney *U* test. Fitness evaluation was rated on a scale from 1 to 6 (1 = bad, 2 = sufficient, 3 = medium, 4 = good, 5 = very good, 6 = competitive sports level).

### 4.1. Cognitive-motor tests

**Figure 5:**
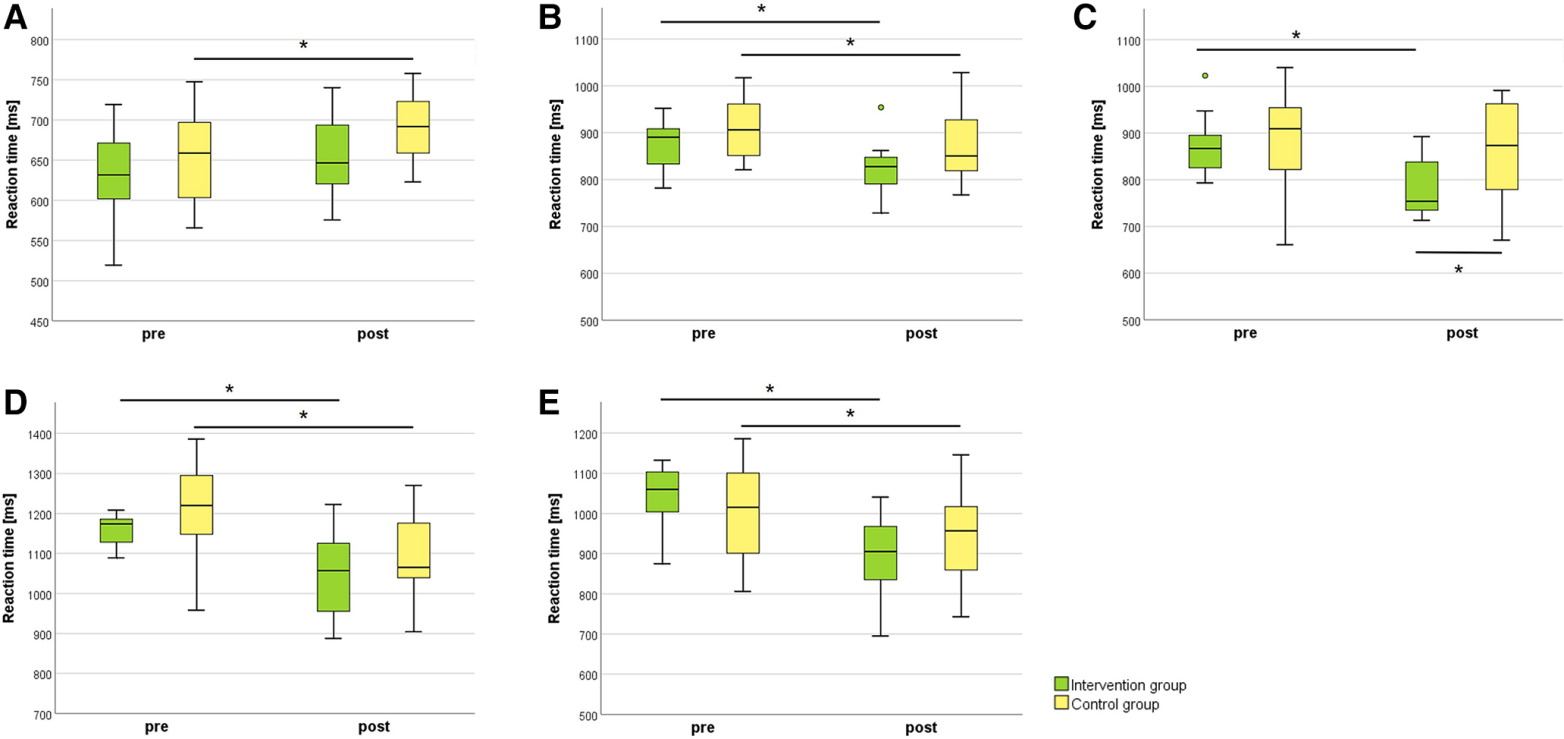
Cognitive-motor test battery results for the intervention and control group comparing pre- and post-intervention measurements. Mean reaction time for (**A**) simple reaction, (**B**) inhibition, (**C**) flexibility, (**D**) divided attention visual, and (**E**) divided attention auditory. *N* = 24; intervention group *N* = 12, control group *N* = 12. *p ≤ 0.05.

Results of the cognitive-motor test battery are displayed in Figure 5 and Table 2. Results showed a significant time main effect (*F* = 6.745, *p* = 0.009, *d* = 1.107) for the simple reaction test. *Post hoc* analysis showed a significant increase (*Z* = −2.510, *p* = 0.012, *r* = 0.72) of the reaction time for the control group (Figure 5A). For inhibition, a significant time main effect (*F* = 20.204, *p* < 0.001, *d* = 1.917) was calculated. *Post hoc* analysis demonstrated a significant decrease of the reaction time for the intervention group (*Z* = −2.589, *p* = 0.010, *r* = 0.75) and the control group (*Z* = −2.040, *p* = 0.041, *r* = 0.59; Figure 5B). For flexibility, results showed a significant time x group interaction effect (*F* = 12.176, *p* < 0.001, *d* = 1.488). *Post hoc* analysis showed a significant decrease of the reaction time for the intervention group (*Z* = −3.059, *p* = 0.002, *r* = 0.88) and a significant difference (*U* = −2.136, *p* = 0.033, *r* = 0.44) of the post-measurement values comparing intervention and control group (Figure 5C). For the divided attention test, two participants of the control group had to be excluded due to technical issues (*N* = 10). For divided attention visual, a significant time main effect (*F* = 38.708, *p* < 0.001, *d* = 2.794) was analysed. *Post hoc* analysis illustrated a significant decrease of the reaction time for the intervention group (*Z* = −2.746, *p* = 0.006, *r* = 0.79) and the control group (*Z* = −2.803, *p* = 0.005, *r* = 0.89; Figure 5D). Moreover, results showed a significant time x group interaction effect (*F* = 9.776, *p* = 0.002, *d* = 1.404) for divided attention auditory. *Post hoc* analysis demonstrated a significant decrease of the reaction time for the intervention group (*Z* = −2.981, *p* = 0.003, *r* = 0.86) and control group (*Z* = −2.395, *p* = 0.017, *r* = 0.76; Figure 5E).

**Table 2:** Results of the pre- and post-intervention measurement comparisons between the intervention and control group for the cognitive-motor test battery.

	Interaction effect (time x group)			Main effect (time)			Main effect (group)		
	*F*	*p*	*d*	*F*	*p*	*d*	*F*	*p*	*d*
Simple reaction	0.869	0.351	0.397	6.745	0.009*	**1.107**	2.129	0.145	0.622
Inhibition	0.512	0.474	0.305	20.204	<0.001*	**1.917**	2.192	0.139	0.631
Flexibility	12.176	<0.001*	**1.488**	28.245	<0.001*	**2.266**	2.206	0.137	0.633
Divided attention visual^a^	0.051	0.821	0.101	38.708	<0.001*	**2.794**	0.956	0.328	0.439
Divided attention auditive^a^	9.776	0.002*	**1.404**	51.314	<0.001*	**3.127**	0.024	0.876	0.070

*N* = 24. Intervention group *N* = 12, control group *N* = 12. Data analysis was performed by using the R package nparLD. Effect size *d* represents a small effect for values between 0.2–0.5, medium effect for 0.5–0.8, and large effect for values over 0.8.

^a^ *N* = 10 (control group).

*p ≤ 0.05.

### 4.2. Alertness test

**Figure 6:**
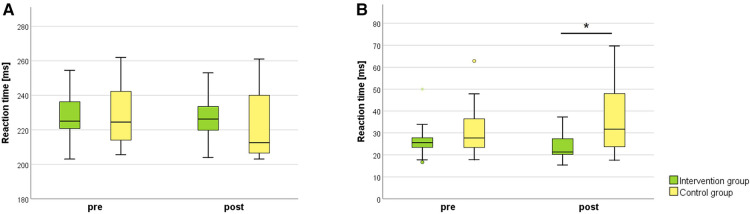
Alertness test for the intervention and control group comparing pre- and post-intervention measurements. (**A**) Median of the reaction time, (**B**) variability of the reaction time. *N* = 24; intervention group *N* = 12, control group *N* = 12. *p ≤ 0.05.

Results of the alertness test performance are displayed in Figure 6 and Table 3. Results showed no significant time x group interaction or time main effects for the median reaction time nor for the variability of the reaction time. A significant group main effect was present for the variability of the reaction time (*F* = 5.277, *p* = 0.022, *d* = 0.980). Between group comparisons resulted in a significant, large effect-sized difference for the post-measurements (*U* = −2.483, *p* = 0.013, *r* = 0.51; Figure 6B).

**Table 3:** Results of the pre- and post-intervention measurement comparisons between the intervention and control group for the alertness test.

	Interaction effect (time x group)			Main effect (time)			Main effect (group)		
	*F*	*p*	*d*	*F*	*p*	*d*	*F*	*p*	*d*
Median of the RT	0.717	0.397	0.361	2.589	0.108	0.686	0.437	0.508	0.282
Variability of the RT	2.196	0.138	0.632	0.073	0.786	0.115	5.277	**0.022** *	0.980

*N* = 24. intervention group *N* = 12, control group *N* = 12. Data analysis was performed by using the R package nparLD. Effect size *d* represents a small effect for values between 0.2–0.5, medium effect for 0.5–0.8 and high effect for values over 0.8. RT = reaction time.

* *p* < 0.05.

### 4.3. Questionnaires

Results of the FSS, PACES and SIMS are presented in Table 4. For FSS, results showed a significant decrease of the score for the items overall (*Z* = −2.586, *p* = 0.010, *r* = 0.53) and absorption by activity (*Z* = −2.823, *p* = 0.005, *r* = 0.58). For the SIMS, results showed a significant increase of the score for the item external regulation (*Z* = −2.756, *p* = 0.006, *r* = 0.56). Furthermore, WEMWBS analysis showed no significant effects (time x group effect: *F* = 0.214, *p* = 0.643, *d* = 0.197; time main effect: *F* = 2.330, *p* = 0.127, *d* = 0.651; group main effect: *F* = 0.522, *p* = 0.470, *d* = 0.308) by comparing pre and post values of the intervention (pre: 56.0 [53.3; 58.0]; post: 53.0 [50.5; 57.3]) and control group (pre: 53.0 [50.0; 58.3]; post 53.0 [46.8; 65.0]).

**Table 4:** Questionnaires about training experience in the intervention group comparing pre- and post-intervention measurements.

		Pre	Post	*Z*	*p*	*r*
FSS	Overall	6.3 (5.7; 6.5)	5.8 (5.2; 6.4)	−2.586	**0.010** *	**0.53**
	Fluency of performance	6.4 (5.7; 6.7)	5.8 (5.5; 6.5)	−1.891	0.059	0.39
	Absorption by activity	6.1 (5.8; 6.5)	5.6 (5.0; 6.3)	−2.823	**0.005** *	**0.58**
	Perceived importance	4.8 (3.3; 5.6)	4.3 (3.5; 5.0)	−1.279	0.201	0.26
PACES		5.9 (5.4; 6.6)	5.7 (5.0; 6.3)	−1.916	0.055	0.39
SIMS	Intrinsic motivation	6.0 (5.8; 6.6)	6.0 (5.3; 6.4)	−0.899	0.369	0.18
	Identified regulation	6.0 (5.5; 6.7)	6.0 (5.7; 6.6)	−0.362	0.717	0.07
	External regulation^a^	2.1 (1.0; 3.1)	3.0 (2.5; 3.9)	−2.756	**0.006** *	**0.56**
	Amotivation^a^	1.6 (1.0; 2.3)	2.0 (1.4; 2.5)	−1.408	0.159	0.29

*N* = 12. Data are median values [interquartile range]. Within group comparisons were analysed using Wilcoxon signed-rank test. FSS, flow short scale, PACES, physical activity enjoyment scale, SIMS, situation motivation scale.

^a^ The higher the scores the better the results, except for (the lower the scores the better).

* *p* ≤ 0.05. *p*-values are two-tailed.

## 5. Discussion

This study was a non-randomized controlled trial examining the training effects of an ExerCube training intervention on cognitive-motor functions, training experience, and mental wellbeing in young team sports athletes. To the best of our knowledge, this is one of the first studies that trained team sports athletes with a fitness game to improve cognitive-motor skills that are important for game play performance.

### 5.1. Cognitive-motor and cognitive abilities

As one of the first studies, this study used a cognitive-motor assessment to test sports-specific abilities in a more ecologically valid setting. Results showed that the intervention group significantly improved the athletes' flexibility and their divided attention (auditive stimuli) cognitive-motor performance (faster reaction time) compared to the control group. These improvements could be explained by the fact that the game environment and mechanism of the Sphery Racer included specific stimuli to trigger improvements in flexibility and divided attention abilities. In terms of flexibility, players had to flexibly switch between the different functional exercises and the individually adaptable real-time scenario. Regarding divided attention, players had to concurrently process the information from the surrounding game environment, the auditory feedback, and the verbal instructions indicating that especially the auditory part is stimulated during the exergame training. For the inhibition and the divided attention (visual stimuli) cognitive-motor assessments, results showed improvements (faster reaction time) for both groups. At this point, one cannot determine how strong the respective impact of the exergame, or sports-specific training regime was on the performance. Thus, results of this study indicate that the athletes were able to transfer certain cognitive-motor abilities from the ExerCube training to the performance of the cognitive-motor assessment.

For the alertness test, no significant results were analysed for the median reaction time. The TAP reference values show that people in the age range of 6–18 years have a median reaction time of 271 ms (47). With values ranging from 203 to 262 ms (min to max; pre, median values) and 203–261 ms (min to max; post; median values), participating athletes already had a fast-processing speed. Thus, not much room for improvement was left, since literature has shown that the fastest reaction time lies between 180 and 200 ms for processing visual stimuli (59). A longer training period, a higher training frequency and a higher training intensity might trigger training effects leading to faster processing times in young athletes who are already on a high-performance level (59). On the other hand, the standard deviation of the reaction time, a critical value considering the stability of the performance (47), showed a significant difference between the groups for the post-intervention measurement in favour of the intervention group. For the time x group interaction effect, a median effect size (*d* = 0.632) was analysed. This improvement could be explained by the fact that athletes had to be physically and cognitively active over 25 min by continuously processing various multisensory game stimuli and executing functional exercises to reach high gaming scores during the exergame training sessions. Previous studies showed that combined training approaches, as exergaming, seem to be particularly advantageous in improving cognitive functions (22, 37, 60).

In summary, exergame training with the ExerCube improved athletes' cognitive-motor performance, especially for flexibility and divided attention (auditive stimuli) abilities as well as their ability to maintain performance on a high level. Improvements in those abilities may enable them to execute early reactions in their sensorimotor system to make their performance more efficient. It can be important for the athletes' field performance and might decide if they win or lose a competition (10). A recent study found a positive association of the cognitive and sports-specific performance domains in youth volleyball and soccer players providing that there is close interplay between cognitive and motor skills in a sports performance context (61). Thus, adding a combined cognitive-motor training approach to the training of athletes would build a more holistic training regime as these couplings are required and highly challenged in real game situations (26). Our training incorporated (motor) responses, visual stimuli, and the perceptual function required when performing the “real-world” task, which are deemed important requirements for such training (62). The environments in similar previous research often lacked the required coupling of perception and action to elicit a true response from the athletes (63), which may explain our favorable results. For exergames to serve as an innovative training approach, it seems important that game environment and mechanics consider the relevant cognitive-motor functions of targeted sports, allowing the training of relevant and different processes in a new training environment (64). Nevertheless, it is still under debate how far the training effects can be transferred to the real sports environment (11, 26).

### 5.2. Training experience and well-being

Regarding training experience, the questionnaire scores are on a medium to high level indicating that the athletes enjoyed the training, were motivated to train, and experienced flow during the training sessions. These results are in line with previous studies showing that the ExerCube triggers beneficial training experiences (38–40, 65). Furthermore, an enjoyable training experience with exergames can help to keep the training motivation and performance on a high level over a longer period (33, 66, 67). Thus, exergames could serve as an enjoyable training addition for athletes allowing them to train important physical and cognitive functions.

Nevertheless, results showed a significant decrease for certain questionnaire items from the first to the last training session. One reason might be the timing of the post-intervention measurement. Due to the COVID-19 restrictions, it was not possible to answer the questionnaires directly after the last training session. Hence, athletes had to retrospectively rate their training experiences, and this might have influenced their rating (52). Another reason might be the excitement about testing new and innovative training approaches. This novelty effect of the first testing of the ExerCube might confound the results and lead to relatively high values. After various training sessions, this novelty effect might have subsided settling the values at a lower level compared to relatively high levels in the beginning.

In terms of well-being, the WEMWBS showed a slight decrease in both groups. It might be that the COVID-19 situation had an influence on the post-intervention measurement values (68). Nevertheless, the median values of both groups were over 51 as in the population sample of the study of Tennant et al., 2007 indicating a good level (54).

### 5.3. Limitations

The presented research is subject to several limitations that must be mentioned. One limitation is the shorter than planned training intervention period. Due to COVID-19 restrictions, the study had to be terminated prematurely. Nevertheless, the results of this study showed, based on the effect size estimates, some promising early indications of a positive effect on EF in young team sports athletes that must be verified in future trials. In these trials, exergame training interventions should be performed over a longer time. Furthermore, the sudden start of the post-intervention measurement due to the COVID-19 restrictions might have had an influence on certain outcomes. Questionnaires (FFS, PACES and SIMS) should have been answered immediately after the last training session allowing direct consideration of the training experience. However, some days were in between the unscheduled “last” training session and filling in the questionnaires. Consequently, the answers of the post-intervention measurement must be treated with caution. Moreover, we must keep in mind that the pandemic situation *per se* might have had an influence on the performance of the post measurements. A further limitation concerns the study design. Athletes were not randomly allocated to the study groups, and this might have led to an allocation bias. However, the baseline data showed no significant differences between the two groups. Because we used convenience sampling, a non-probability sampling method where units are selected for inclusion in the sample because they are available at a given time, and show willingness to participate, we cannot generalize our findings to other less active young athletes. Future studies, nevertheless, should consider implementing a random study allocation procedure to minimise study design bias and to increase the validity of the results. Another limitation that affected the generalisation of these results is the sample size. The study included a convenience sample of young athletes from different field sports, with only a small number of participants per discipline. As a result, future studies should consider performing intervention studies with a larger sample size per sports discipline to increase the generalizability of the results to these specific sports.

## 6. Conclusion

This study is one of the first studies indicating that exergaming has positive effects on cognitive-motor interactions, especially on concentration, flexibility, and divided attention, in young team sports athletes. Thus, exergaming, if designed properly, can be an innovative complementary training approach for team sports athletes as these couplings of cognitive and motor functions are required and highly challenged in real-life game situations. Furthermore, exergames could serve as a motivating training for the young athletes triggering relevant stimuli in a new training environment to the young athletes. Further exergame design explorations and studies are needed to examine the effects of exergaming on sports-specific performance and to define specific exergame design requirements for athletes.

## Data Availability

The original contributions presented in the study are included in the article/Supplementary Material, further inquiries can be directed to the corresponding author.
